# Poly(*N*-vinylcaprolactam)–Gold Nanorods–5 Fluorouracil Hydrogels: In the Quest of a Material for Topical Therapies against Melanoma Skin Cancer

**DOI:** 10.3390/pharmaceutics15041097

**Published:** 2023-03-29

**Authors:** Mirian A. González-Ayón, Alondra Rochin-Galaviz, Arturo Zizumbo-López, Angel Licea-Claverie

**Affiliations:** Centro de Graduados e Investigación en Química, Tecnológico Nacional de México/Instituto Tecnológico de Tijuana, Apartado Postal 1166, Tijuana 22454, Mexico

**Keywords:** *N*-vinylcaprolactam, hydrogels, gold nanorods, 5-fluorouracil, controlled drug release

## Abstract

Chemically crosslinked hydrogels based on poly(*N*-vinylcaprolactam) (PNVCL) were synthetized by a photoinitiated chemical method. A galactose-based monomer, 2-lactobionamidoethyl methacrylate (LAMA), and *N*-vinylpyrrolidone (NVP) were added with the aim to improve the physical and chemical properties of hydrogels. The effects of both comonomers on the swelling ratio (Q), volume phase transition temperature (VPTT), glass transition temperature (T_g_), and Young’s moduli by mechanical compression below and above the VPTT were studied. Gold nanorods (GNRDs) and 5-fluorouracil (5FU) were embedded into the hydrogels, to study the drug release profiles with and without the excitation of GNRDs by irradiation in the near-infrared region (NIR). Results showed that the addition of LAMA and NVP increased the hydrogels’ hydrophilicity, elasticity, and VPTT. The loading of GNRDs in the hydrogels changed the release rate of 5FU when irradiated intermittently with an NIR laser. The present study reports on the preparation of a hydrogel-based platform of PNVCL-GNRDs−5FU as a potential hybrid anticancer hydrogel for chemo/photothermal therapy that could be applied against skin cancer for topical 5FU delivery.

## 1. Introduction

Skin cancer is one of the most common human malignancies with a high incidence rate around the world [1]. Specifically, cutaneous melanoma is the most aggressive and deadliest skin cancer. The American Cancer Society anticipates 97,610 patients with melanoma in 2023, with an estimated 7990 deaths [2]. It originates from pigmented cells and arises from cells after neoplastic transformation into spindle-like, small, and epithelioid melanocytes, also containing melanin granules [3]. Melanoma can easily spread or metastasize, which leads to difficulties in skin cancer treatment and often results in the high mortality of patients.

5-Fluorouracil (5FU) is an anticancer drug that is widely used in chemotherapy against melanoma skin cancer. This drug can be administered orally or intravenously. However, the conventional administration of 5FU, on the one hand, causes severe systemic side effects due its cytotoxic effect on normal cells, and, on the other hand, it has a short biological half-life due to rapid metabolism and incomplete and non-uniform oral absorption due to rapid degradation by dihydropyramidine dehydrogenase [4].

In order to substantially improve the delivery of antineoplastic agents, polymeric matrices with different morphologies have been studied [5,6]. Polymers with a network structure, at nano- and microscopic scales, have shown important characteristics for the absorption and controlled release of drugs [7]. However, due to the skin’s poor retention, conventional topical dosage forms appear ineffective in treating these conditions. Based on patient compliance, safety, efficacy, feasibility, and self-life, controlled topical drug delivery systems have gained popularity in recent decades.

Hydrogels have been widely used as wound dressings due their ability to incorporate a large volume of water into their structure, behaving more similarly to living tissue than any other synthetic material. Additionally, through control over its physicochemical properties, it is possible to significantly influence its drug loading and release capacity, and in vitro and in vivo stability [8]. In particular, temperature-responsive hydrogels have been extensively studied for biomedical and pharmacological applications, mainly because they can reversibly change from a swollen to a shrunken state at a specific temperature, known as the volume phase transition temperature (VPTT). Poly(*N*-vinylcaprolactam) (PNVCL) is a thermosensitive and biocompatible polymer. Studies have shown that PNVCL may inhibit some strains of pathogenic bacteria, such as *Escherichia coli* (Gram negative) [9], *Staphylococcus aureus* (Gram positive) [10], and the yeast *Candida albicans* [11]; therefore, it is a polymer of great interest for biomedical applications [12].

Hydrogels made of carboxymethylcellulose, chitosan, bovine serum albumin, and other biopolymers are widely considered for drug delivery systems [13]. Natural polymers could improve biodegradation, biocompatibility, cytocompatibility, and long-term release. Specifically, galactose-based polymers are of great interest in cancer treatment, due to their affinity to overexpressed asialoglycoprotein (ASGP-R) in liver cancer [14] and to the prometastatic protein galectin-3’s overexpression in prostate cancer [15], colon cancer [16], breast cancer [17], hemangiosarcoma [18], multiple myeloma [19], and melanoma [20].

In addition, subsequent advances have demonstrated the prospective use of hybrid hydrogel systems as intelligently designed architectures built by the integration of polymer(s) and drug–metal particle conjugates. Recently, some studies have shown that the combination of gold nanorods (GNRDs) and a chemotherapeutic agent results in synergistic anticancer efficacy in in vitro and in vivo models [21].

**Figure 1:**
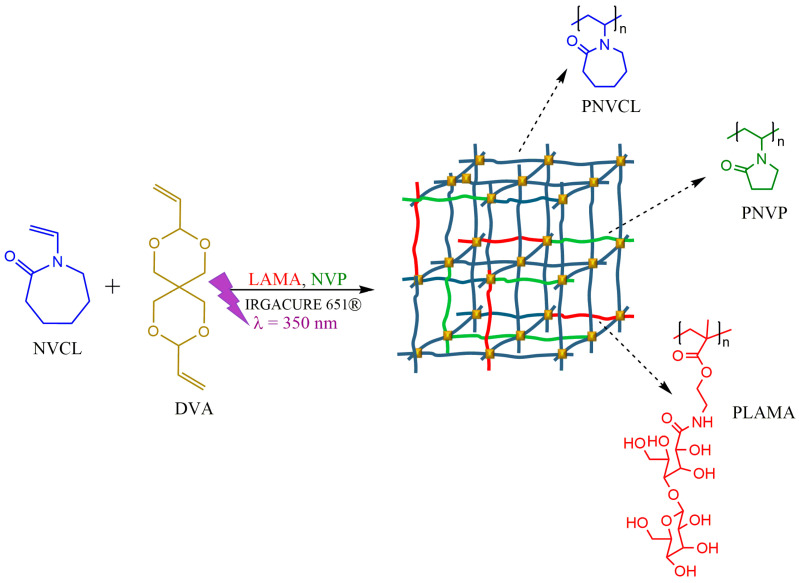
Scheme of the preparation of NVCL-based hydrogels via photopolymerization.

In this work, PNVCL hydrogels chemically cross-linked via photopolymerization are reported (Figure 1). 

Many techniques for the synthesis of hydrogels have been reported with success; however, photopolymerization offers several advantages over conventional polymerization methods. These include spatial and temporal control over the polymerization, fast curing times, and minimal heat production [22]. A galactose-based monomer (LAMA) and NVP were added with the aim to improve the physical and chemical properties. The effects of both comonomers on the swelling ratio (Q), VPTT, glass transition temperature (T_g_), and mechanical compression behavior below and above the VPTT were assessed. The Franz diffusion cell method was used to determine the in vitro drug release of 5FU loaded in the hydrogels together with GNRDs. Under humidity and thermal control, the developed hydrogels could achieve the sustained release of the loaded drug. The kinetics of drug release in the absence and presence of NIR irradiation were compared with kinetic models. The prepared hydrogel nanocomposite formulations were evaluated as candidate biomaterials with desired properties in the search for new skin cancer treatment methods.

## 2. Materials and Methods

### 2.1. Materials

*N*-vinylcaprolactam (NVCL, 98%) was purified by recrystallization in hexane and dried under a vacuum prior to use. In this study, 3,9-divinyl-2,4,8,10 tetra-oxaspiro [5.5] undecane (DVA, 98%), 2,2-dimethoxy-2-phenylacetophenone (IRGACURE 651^®^, 99%), *N*-vinylpyrrolidone (NVP, 99%), 5-fluorouracil (5FU, 99%), cetyltrimethylammonium bromide (CTAB, 99%), gold (III) chloride hydrate, sodium borohydride (NaBH_4_, 99%), silver nitrate (AgNO_3_, 99%), sulfuric acid (H_2_SO_4_, 98%), and ascorbic acid (AA, 98%) were used as received. All deuterated solvents and reagents were purchased from Sigma-Aldrich (Toluca, Mexico). The galactose-based monomer, 2-lactobionamidoethyl methacrylate (LAMA), was synthesized following a procedure reported previously [23]. Gold nanorods (GNRDs) were synthesized in an aqueous CTAB solution using a seed growth method, as reported in the literature [24,25]. Ethanol was provided by Fermont (Monterrey, Mexico) and distilled water by Sparkletts (Lakeside, CA, USA).

### 2.2. Preparation of NVCL-Based Hydrogels by Photopolymerization Method

PNVCL crosslinked hydrogels were obtained by photopolymerization, using a photochemical reactor (Rayonet model RPR-200, Palisades Park, NJ, USA) with ultraviolet lamps of wavelength 350 nm. To obtain PNVCL hydrogels, NVCL (7 g, 50 mmol), DVA (0.213 g, 1 mmol), and IRGACURE 651^®^ (0.111 g, 0.43 mmol) were dissolved in a mixture of water/ethanol (47/53 vv%) in a Schlenk flask at room temperature. The mixture was bubbled with nitrogen for 10 min, in order to remove any oxygen present. Then, the precursor solution was transferred into a glass mold, which consisted of two plates of glass separated by a 2-mm-thick silicone spacer; after injection, the silicone was sealed to avoid the entrance of oxygen. Subsequently, the filled mold was exposed to ultraviolet (UV) light at a wavelength of 350 nm for 1 h. Similarly, hydrogels with different percentages of LAMA, NVP, or both monomers were obtained. The content of LAMA monomer was modified at 1, 2, and 3 mol% with respect to NVCL, while NVP was varied at 5, 10, and 15 mol% with respect to NVCL. In all cases, the obtained hydrogel sheets were removed from the mold and were purified by extraction with ethanol/water mixtures (75/25, 50/50, and 25/75 by volume) with changes of fresh mixtures every day. After purification, the still wet hydrogel sheets were cut into discs with diameters of 10 mm. Then, the discs were dried in a vacuum oven at 65 °C for 24 h.

### 2.3. Characterization

#### 2.3.1. Swelling Analysis and VPTT Determination

The swelling behavior of hydrogels was studied by measurements of *Q* at equilibrium (Equation (1)), assuming isotropic swelling. The dry hydrogel samples (discs) were weighted (*W_d_*). Then, the discs were placed in a glass container with 50 mL of water at 25 °C for 48 h and the swelled hydrogels discs were taken out at different times. The swelled hydrogel was wet weighed (*W_s_*), after tapping out excess liquid.

(1) $$Q=\frac{V_{s}}{V_{d}}=1+\frac{W_{sol}\rho_{d}}{W_{d}\rho_{sol}}$$

where *V_s_* is the volume of swelled hydrogel, *V_d_* is the volume of dry hydrogel, *W_sol_* is the weight of the absorbed solvent (*W_sol_* = *W_s_* − *W_d_*), *ρ_sol_* is the density of the solvent (in this case, water), and *ρ_d_* is the density of the dry hydrogel. The *ρ_d_* used in all cases was 1.23 g cm^−3^, as reported in the literature for PNVCL hydrogels [26].

The VPTT was determined by swelling experiments at different temperatures. For this, a double-jacketed reaction beaker connected to a thermostatic water bath at 5 °C was used. A disc from each hydrogel was placed into 50 mL of deionized water for 24 h, and then the sample was weighted for Q determination by Equation (3) at 5 °C. Then, the temperature was increased in 5 °C steps and the samples were left to stand for 24 h at each temperature, recording the wet hydrogel weight, until reaching 55 °C. From the graph of Q as a function of temperature, VPTT was considered as the intersection between the shrinking tangent and the tangent where Q stabilized its value at a high temperature.

#### 2.3.2. Thermal Analysis by DSC and TGA

The T_g_ of hydrogels was obtained by using differential scanning calorimetry (DSC) equipment (TA Instruments, Model Q2000, New Castle, DE, USA). Modulated DSC mode was employed and the measurements were performed using dry hydrogel powder (10 mg). The method comprised two temperature cycles; in the first one, each sample was cooled to −30 °C, maintained isothermally for 5 min, the temperature was modulated to ± 0.5 °C every 60 s, and the sample was heated with a ramp of 10 °C min^−1^ up to 200 °C. For the second cycle, the heating ramp was changed to 5 °C min^−1^ up to 200 °C.

The decomposition temperature was measured by thermogravimetric analysis equipment (TGA) from TA Instruments (Discovery Model, New Castle, DE, USA), using a heating ramp of 10 °C min^−1^ from 25 °C to 600 °C in a nitrogen atmosphere. The weight loss and weight residue were recorded during the heating ramp.

#### 2.3.3. Mechanical Analysis

Dynamic mechanical analysis (DMA) was performed in DMA Q800 equipment from TA Instruments (New Castle, DE, USA). Samples in the form of discs with a diameter of 10 mm were previously swelled for 24 h in distilled water. The measurements were carried out at 25 °C and 37 °C using a compression test fixture with submersion in distilled water. Compression tests were evaluated for each type of material in triplicate.

#### 2.3.4. 5FU and GNRDs Loading into Hydrogels

To investigate the 5FU and GNRDs loading capacity and the controlled drug release from hydrogels, the equilibrium swelling method was used. The method was carried out as follows: a dry hydrogel disc with 1 cm diameter was placed into a loading solution containing 5FU and GNRDs. The preparation of the loading solution consisted of two parts: on the one hand, a 4 mL solution of 5FU in water (10 mg mL^−1^) was prepared; on the other hand, 10 mL of CTAB-stabilized GNRDs dispersion was purified to eliminate CTAB by two cycles of centrifugation at 10,000 rpm, eliminating the supernatant and re-dispersing it in pure water. The thus-purified GNRDs were concentrated in 5 mL of water. Then, the two previous solutions were combined, and 1 mL of water was added, obtaining 10 mL of loading solution. Then, dried hydrogels were placed in single vials and 1 mL of the loading solution was added to each one. They were kept covered from light and stored at 15 °C for 48 h. In the case of the hydrogels that would be irradiated in the near-infrared region during the release experiments, the loading was carried out in the same way, only modifying the storage temperature, which was 4 °C, to allow greater swelling of the hydrogel and achieve higher absorption of GNRDs in the polymer network. After this, the discs were washed with water to remove surface-adsorbed 5FU and GNRDs. The supernatant was assayed spectrophotometrically for non-absorbed 5FU content using a UV–Vis, Varian-Cary 100 spectrophotometer (Agilent Technologies, Santa Clara, CA, USA) at the wavelength of 266 nm. The drug loading (%DL) and encapsulation efficiency (%EE) were calculated using Equations (2) and (3), respectively.

(2) $$\%\mathrm{DL}=\left(\frac{{\mathrm{MD}}_{h}}{\mathrm{hydrogel}\;\mathrm{mass}\;+{\mathrm{MD}}_{h}}\right)\times 100$$

(3) $$\%\mathrm{EE}=\left(\frac{\mathrm{initial}\;\mathrm{MD}-\mathrm{supernant}\;\mathrm{MD}}{\mathrm{initial}\;\mathrm{MD}}\right)\times 100$$

where MD_h_ is the mass of drug in the hydrogel (5FU in the loading solution minus 5FU in the supernatant) and MD is the mass of drug.

On the other hand, the GNRDs loading was determined by TGA. For this, the residue at 550 °C of GNRDs-loaded hydrogel samples measured in a nitrogen atmosphere was subtracted from the residue measured at the same temperature for non-GNRDs-loaded hydrogels. The TGA equipment and method used are described in Section 2.3.2.

#### 2.3.5. 5FU In Vitro Release Studies

In vitro 5FU release studies were carried out under sink conditions in a Franz diffusion cell with a slight modification. In the Franz diffusion cell, the receptor volume was 60 mL and 1 cm^2^ was the diffusion area. The hydrogel samples were cut to the appropriate size to be placed in the donor compartment and placed in contact with the phosphate-buffered saline (PBS) receptor (pH 5) at 37 °C. The tests were performed in duplicate. The temperature of the outer jacket was adjusted to 37 °C and kept constant using a thermostat. All analyses were conducted under similar conditions. At predetermined time intervals, an aliquot (1 mL) was withdrawn, and 1 mL of clean PBS was added to keep the volume of release medium constant. The drug content in the aliquot was determined by UV–Vis spectroscopy by the same method described before for drug loading determination. The percentage of drug release (%DR) was calculated by Equation (4):

(4) $$\%\mathrm{DR}=\left(\frac{\mathrm{mass}\;\mathrm{of}\;\mathrm{drug}\;\mathrm{released}\;\mathrm{from}\;\mathrm{hydrogel}}{\mathrm{mass}\;\mathrm{of}\;\mathrm{total}\;\mathrm{drug}\;\mathrm{loaded}\;\mathrm{in}\;\mathrm{the}\;\mathrm{hydrogel}}\right)\times 100$$

The same methodology was employed for the release experiments where NIR irradiation was used. In hydrogels loaded with 5FU and GNRDs, intermittent irradiation was applied. Samples were irradiated with a 808 nm laser (μLS Micro Laser Systems, 450 W, Model: FI3-808-450-FC, CA), during the first 5 min of every hour, for 4 h. Then, after 1 h, an aliquot (1 mL) was withdrawn, and 1 mL of clean PBS was added to keep the volume of release medium constant. The drug content in the aliquot was determined by UV–Vis spectroscopy, as described before.

## 3. Results

### 3.1. Swelling Behavior and Phase Transition Temperature of PNVCL Hydrogels

The photoinitiated polymerization of *N*-vinylcaprolactam-based hydrogels was achieved for various formulations, which consisted of either LAMA or NVP as comonomers in the presence of DVA as a crosslinking agent and IRGACURE 651^®^ as an initiator. The effect of the concentration of comonomers on the hydrogel properties was also investigated. LAMA and NVP were chosen as comonomers due to their hydrophilicity and potential to increase the VPTT of the hydrogel, in addition to their good solubility in water. Polymerization studies were performed for different concentrations of LAMA and NVP added individually, and for both mixed with NVCL, as shown in Table 1. The nomenclature, used according to the feed, is the following: HG-NVCL (H), HG-NVCL-LAMA_1wt%_ (HL1), HG-NVCL-LAMA_2wt%_ (HL2), HG-NVCL-LAMA_3wt%_ (HL3), HG-NVCL-VP_5mol%_ (HV5), HG-NVCL-VP_10mol%_ (HV10), HG-NVCL-VP_15mol%_ (HV15), HG-NVCL-LAMA_3wt%_-VP_5mol%_ (HL3V5), HG-NVCL-LAMA_3wt%_-VP_10mol%_ (HL3V10), and HG-NVCL-LAMA_3wt%_-VP1_5mol%_ (HL3V15).

**Figure 2:**
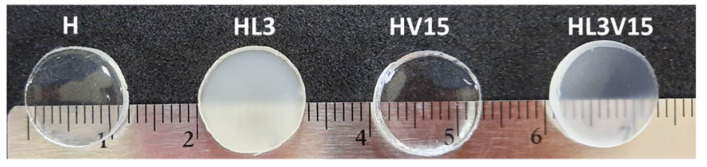
Photographs of NVCL-based hydrogel discs swollen in water.

PNVCL hydrogel (H) samples were transparent, indicating that the polymers were amorphous, with chains distributed in a random fashion (Figure 2). This was further supported by DSC analysis, with samples exhibiting an endothermic transition associated with the T_g_ characteristic of PNVCL, as discussed later. Hydrogels containing NVP showed a similar appearance to those containing NVCL only, while those containing LAMA in their structure showed an opaque white coloration (Figure 2).

**Figure 3:**
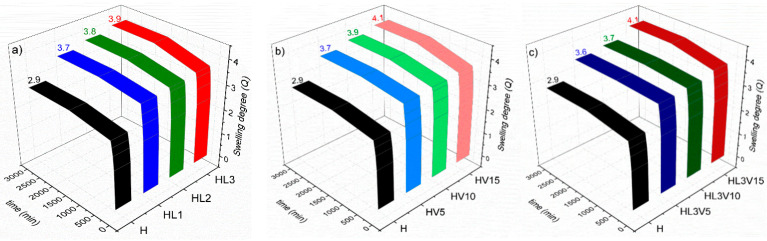
Equilibrium swelling profiles at room temperature (~25 °C) for NVCL hydrogels: (**a**) with LAMA, (**b**) with NVP and, (**c**) with LAMA and NVP (the hydrogel without LAMA/NVP, named H, is shown in each graph for comparison).

The time required to reach the maximum degree of swelling or Q was analyzed by continuous measurements over 48 h, as shown in Figure 3. The results show that equilibrium swelling was achieved after 24 h of water submersion for all hydrogels. In this sense, with the objective of analyzing the thermal sensitivity of the hydrogels, a swelling analysis at different temperatures was also performed.

**Figure 4:**
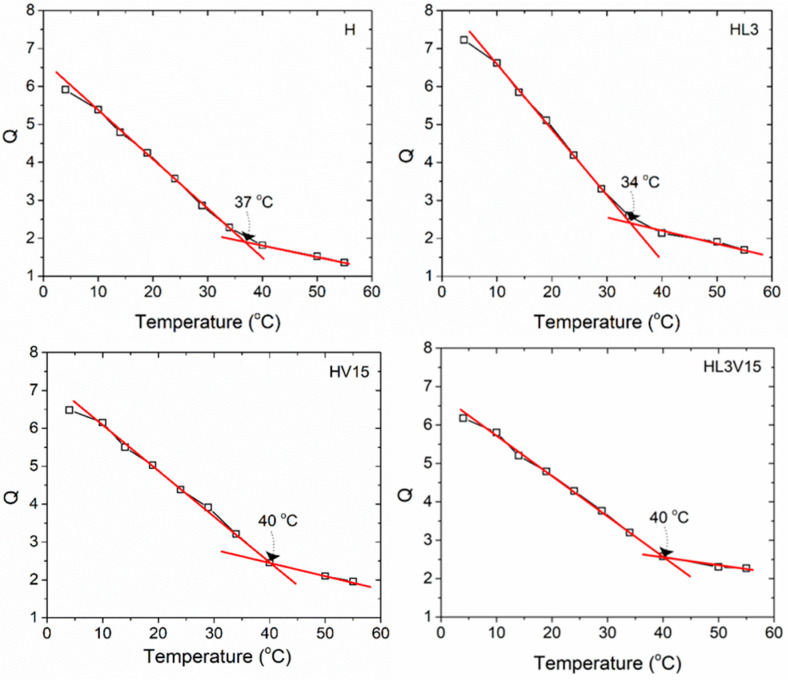
Equilibrium swelling degree (Q) of H, HL3, HV15, and HL3V15 at different temperatures ranging from 5 to 55 °C.

As shown in Table 1, the Q-values indicate that the materials have a good capacity to absorb water, increasing the weight, at equilibrium, from 2 to 4 times its dry weight. Q-values for all hydrogels are higher at 25 °C compared to those measured at 37 °C. This tendency is attributed to the thermoresponsive character of PNVCL, which is based on the lower critical solution temperature (LCST) of linear PNVCL chains. PNVCL homopolymers of different molecular weights exhibit an LCST from 30 to 38 °C [27]. At high temperatures, the polymer chains break the hydrogen bonds with water molecules, expelling water from the structure and favoring ring-to-ring interactions of the hydrophobic caprolactam ring [28]. In the hydrogel morphology, this results in the shrinkage of the PNVCL network, leading to lower values of Q. This behavior is represented as the VPTT, which could be controlled through the adjustment of the hydrophilicity and hydrophobicity of the polymer chains in the hydrogel network. By altering the feed ratio of LAMA and/or NVP monomers, samples were synthesized to have their own phase transition temperatures, as shown in Table 1 and Figure 4. The swelling behavior of the NVCL-based hydrogels at different temperatures is shown in Figure 4 and Figures S1–S6 (in the Supplementary Materials). For all samples, as the temperature increased from 5 to 55 °C, the degree of swelling decreased markedly. It was possible to obtain NVCL-based hydrogels with a VPTT between 37 and 40 °C. On the one hand, the presence of the LAMA monomer in the hydrogel decreased slightly the VPTT, and allowed relatively higher swelling degrees. On the other hand, the presence of NVP increased the VPTT from 37 to 40 °C, also causing a higher degree of swelling on the hydrogels. The higher Q-values are attributed to the fact that both LAMA and NVP have a more hydrophilic character than NVCL, which, at low temperatures, allows a better interaction between the polymer network and the water, through hydrogen bonds, leading to higher water content in the polymer network and therefore to the disentanglement of the polymer chains.

As shown in Figure 4, although, at 55 °C, the swelling of the hydrogels decreased considerably, they still contained water in the network. The water content at this temperature was higher for hydrogels containing LAMA and/or NVP as compared to the pure NVCL hydrogel (H). This is attributed to the greater presence of hydrogen-bond-type interactions between the amide, ester, and hydroxyls of LAMA, as well as by the carbonyl of NVP and NVCL with water molecules, resulting in the fact that not all hydrogen bonds to water are broken by calorific energy up to 55 °C.

### 3.2. Dry Hydrogel Behavior and Glass Transition Temperature Analysis of NVCL-Based Hydrogels

**Figure 5:**
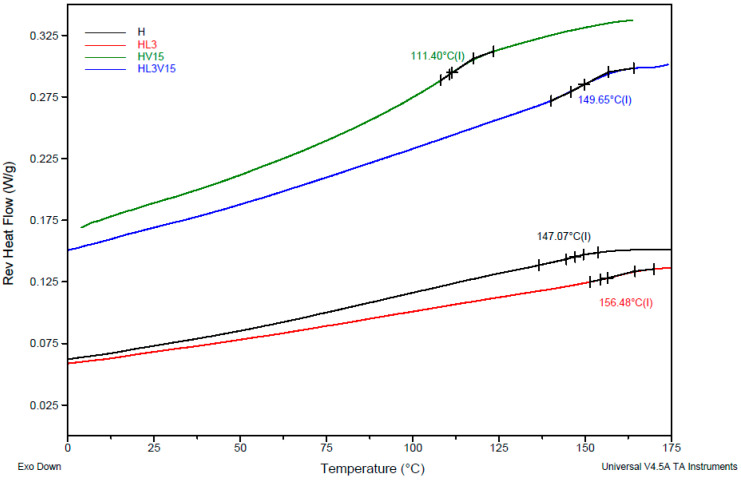
DSC thermograms for NVCL-based hydrogels with LAMA and/or NVP.

The influence of LAMA and/or NVP content on the thermal behavior of dry PNVCL-based hydrogels was investigated by means of DSC. Figure 5 shows an example of a thermogram for hydrogels containing NVCL (H), NVCL with LAMA (HL3), NVCL with NVP (HV15), and NVCL with both LAMA and NVP (HL3V15). The T_g_ values are indicated for each type of hydrogel. Hydrogel H shows a T_g_ value of 147 °C, typical for an NVCL-based polymer network [28]. In hydrogels containing LAMA, the results show an increase in T_g_ values from 108 to 137 and to 156 °C, while the LAMA content increases (Figure S7 in the Supplementary Materials). When NVP was copolymerized with NVCL, the T_g_ value of the hydrogels was decreased from 116 to 111 °C, when the NVP content increased from 5 to 15 mol% in the recipe (Figure S8 in the Supplementary Materials), precluding viscoelastic properties. Finally, in NVCL hydrogels containing 3 mol% LAMA and varying NVP content of 5, 10, and 15 mol%, the results for T_g_ were 147, 135, and 149 °C. In all systems, the results correspond well with the expectations for the incorporation of LAMA in the polymer network, since the higher content of these comonomers, the greater number of intermolecular interactions of the polymeric network, and therefore the greater impediment of chain movements in the network, so that the glass transition is shifted to higher temperatures, precluding a more brittle material.

### 3.3. Young’s Moduli of Hydrogels at Different Temperatures

**Figure 6:**
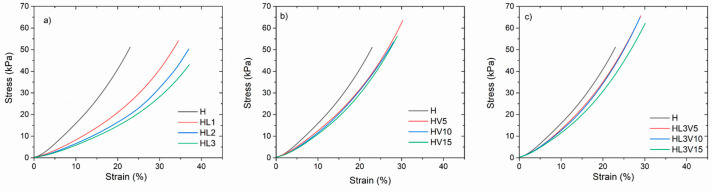
Stress–strain curves for compression under submersion in water at 25 °C, for PNVCL hydrogels containing LAMA (**a**), NVP (**b**), and LAMA and NVP (**c**).

The improvement of the mechanical properties, to obtain more elastic materials of PNVCL-based hydrogels by including LAMA and or NVP as copolymers, was one of the main goals of this work. The potential application sought for PNVCL hydrogels is as biomaterials, taking advantage of the properties described in the Introduction section; therefore, the mechanical properties of interest related to the wet (swollen) state. Stress–strain curves for compression submerged in water at a temperature below the VPTT (25 °C) (Figure 6) and above or at the VPTT (37 °C) (Figures S9–S11 in Supplementary Materials) are presented. The data of moduli and deformation as a result of mechanical compression are summarized in Table 2. In general, the results at 25 °C show that when LAMA and/or NVP are incorporated in the polymer network, as well as when the comonomer content increases, the Young´s modulus decreases and the maximum deformation increases. All hydrogels are more rigid when they are at 37 °C, which is attributed to the fact that, at 25 °C, hydrogels are mostly swollen in water, so they are softer materials compared to the same materials at 37 °C, where a large part of the water is expelled from the polymeric network by breaking the hydrogen bonds between the polymer and the water. At this point, intermolecular interactions are favored and therefore the network becomes more compact or rigid, giving rise to higher Young´s moduli compared to those obtained at 25 °C.

Hydrogels containing LAMA (HL1, HL2, HL3) were found to be the more elastic hydrogels, showing higher deformation, while the VPTT was shifted to lower values (Table 1). For a biomedical and/or pharmaceutical application, the hydrogels copolymerized with NVP or both LAMA-NVP, showing VPTT values between 37 and 40 °C, are the most promising. In this sense, the H, HL3, HV15, and HL3V15 hydrogels were selected to evaluate their capacity for the loading of 5FU and GNRDs and to determine the kinetics of drug release under physiological skin conditions (37 °C and pH 5) with and without NIR irradiation.

### 3.4. In Vitro Drug Release Studies

#### 3.4.1. Photothermal Analysis of PNVCL Hydrogels with GNRDs

**Figure 7:**
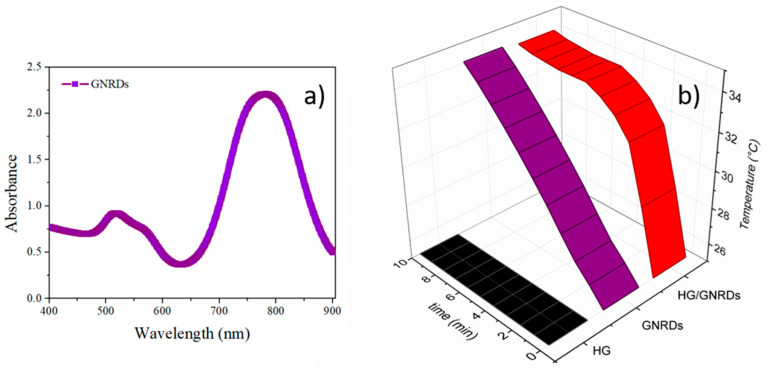
(**a**) UV–Vis spectrum showing the localized surface plasmon resonance of gold nanorods and (**b**) in situ temperature increase by NIR irradiation of GNRDs and GNRDs loaded in hydrogel H.

Gold nanorods and 5FU were encapsulated into the NVCL-based hydrogels (H, HL3, HV15, and HL3V15). First, GNRDs in water were characterized by UV–Vis spectroscopy (Figure 7a) and by transmission electron microscopy (Figure S12 in the Supplementary Materials), and the results show two absorption bands, one transversal at 520 nm and the last one at 800 nm corresponding to the longitudinal section of the nanorod evolving from its localized surface plasmon resonance (LSPR). The encapsulation of GNRDs in the hydrogels was also verified via irradiation with an 808 nm NIR laser. The results show a clear increase in the local temperature of around 10 °C (Figure 7b), similar to the temperature increase under irradiation of the pure GNRDs, while the irradiation of the empty hydrogel H is reported as a control. According to the literature, the irradiation of GNRDs having an LSPR at around 800 nm with an NIR laser of 808 nm could increase the local temperature to produce thermal ablation with temperatures above 47 °C or to encourage the susceptibility of cancer cells (sensitization in temperature range 41–45 °C). An elevated tissue temperature increases vascular permeability, increases blood flow, and mitigates tissue hypoxia [29]. Since hyperthermia can increase the sensitivity of cancer cells to chemotherapy drugs, its joint application intensifies the cytotoxic effects in the tumor, so the combination of these two modalities can improve the efficacy of cancer treatment [30].

#### 3.4.2. Franz Diffusion Drug Delivery

The transdermal delivery mechanism for drug administration has been generally utilized to treat disease conditions. This system has the objective of delivery to the target site through various skin layers into the bloodstream, with little or no systemic circulation, as well as to protect the drug and increase its biological half-life.

According to previous studies, the normal pH of the human skin is around pH 5 and the normal body temperature is between 36.5 and 37 °C, while, in cancer tissue, the pH and temperature are ~6 and ~40 °C, respectively [3,7,25]. Therefore, in vitro 5FU release studies were carried out by mimicking the physiological conditions of healthy skin (pH 5 and 37 °C) and through hyperthermia, employing the NIR irradiation of systems to produce a local temperature increase. A phosphate buffer of pH 5 (0.1 mM) and an 808 nm laser were used.

**Figure 8:**
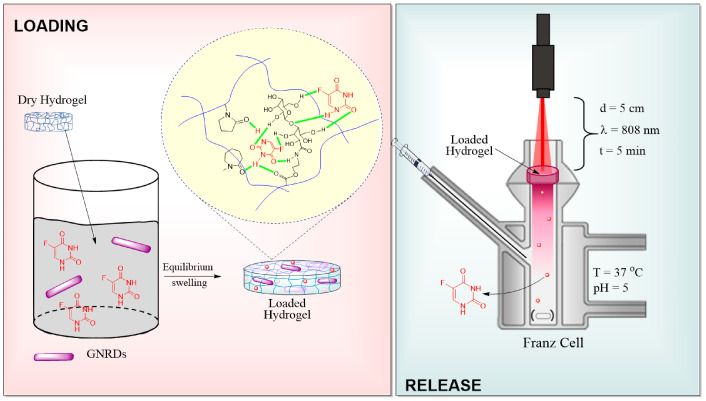
Schematic illustration of 5FU and GNRDs loading in PNVCL-based hydrogels and schematic of the 5FU release experiments using an NIR laser.

The loading content of 5FU in hydrogels H, HL3, HV15, and HL3V15 was between 3 and 5 wt%, whereas the encapsulation efficiency was within the range of 47 to 53 wt% (Table 3). The encapsulation capacity is attributed to the formation of hydrogen bonds and electrostatic forces between 5FU and the amide group in NVCL and/or the ester group in NVP and LAMA in the polymer network, as schematized in Figure 8.

**Figure 9:**
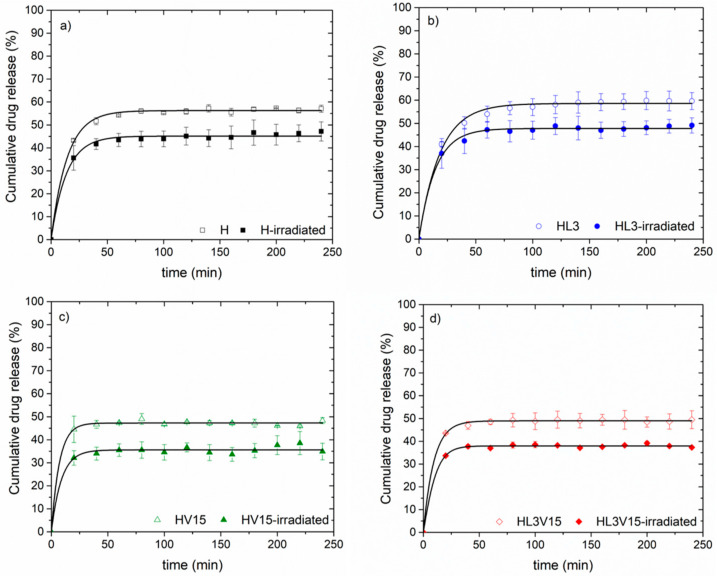
Cumulative 5FU release from NVCL-based hydrogels with and without NIR irradiation: (**a**) H, (**b**) HL3, (**c**) HV15 and (**d**) HL3V15 hydrogels.

On the other hand, the GNRDs loading capacity in the hydrogels was estimated by thermogravimetric analysis (Figures S13–S20 in the Supplementary Materials), showing values between 3 and 6 wt%. GNRDs loading was confirmed by in situ photothermal assays employing an 808 nm laser, as shown in Figure 7. Figure 9 shows the cumulative release profiles of 5FU without and with irradiation through the selected hydrogels (H, HL3, HV15, and HL3V15). From trials at 37 °C (without irradiation), it was demonstrated that hydrogels with a lower VPTT, such as HL3 (VPTT = 34 °C) and H (VPTT = 37 °C), presented higher release rates compared to those with a VPTT = 40 °C (HV15 and HL3V15). This behavior could be attributed to the network shrinkage in the case of hydrogels H and HL3, expelling more 5FU through the breakage of hydrogen bonds between the polymer and drug; meanwhile, for hydrogels HV15 and HL3V15, the VPTT was not reached during the experiment. In all cases, when the system was irradiated at 808 nm during the first 5 min of every hour, the percentage of drug released was lower compared to the same system without irradiation. This behavior can be attributed to the fact that irradiation can increase the temperature of the system above its VPTT, causing the formation of a skin layer, which decreases the diffusion of the drug through the polymer matrix. In this sense, by achieving the slower release of the drug, one could allow longer and, at the same time, more efficient therapies, due to the sensitization of cancer cells by NIR irradiation [31,32]. Drug diffusion from hydrogels at 37 °C and pH 5 without irradiation was faster than the same systems at 37 °C and pH 5 with NIR irradiation, for 5 min every hour. This highlights the control that can be achieved with these engineered thermosensitive materials.

The 5FU release kinetics were studied through different mathematical models (Equations (5)–(8)). Zero-order, first-order, Higuchi, and Peppas models were employed [33].

(5) $$\mathrm{Zero}\;\mathrm{order}\;M_{t}=M_{0}+k_{0}t$$

(6) $$\mathrm{First}\;\mathrm{order}\;F=1-e^{-kt}$$

(7) $$\mathrm{Higuchi}\;F=k\;t^{1/2}$$

(8) $$\mathrm{Peppas}\;F=kt^{n}$$

where *F* is the fractional drug release, *M_t_* is the mass of drug at time *t*, *M*_0_ is the initial mass of drug in the solution, *k* is the release rate constant for different equations, and *n* is the diffusional exponent.

The results of fitting experimental data to mathematical models are presented in Table 4. The zero-order, first-order, Higuchi, and Peppas models were applied to determine the in vitro 5FU release kinetics, at pH 5 and 37 °C, in systems with and without irradiation (808 nm). NVCL-based hydrogels help in the controlled and sustained release of 5FU. The swelling and de-swelling processes also affect the 5FU release kinetics. The release rate (k) and the regression coefficient (r^2^) were obtained by the fitting of the models, as shown in Table 4. In general, the most appropriate models for adjustment were selected according to linearity (r^2^). The best fitting for 5FU release corresponded to the Higuchi and Peppas models when the hydrogel release experiments were performed at a temperature higher than the corresponding VPTT. In the two cases where the release temperature was below the VPTT, e.g., HV15 and HVL3V15 not irradiated, the best-fitting model was the zero-order kinetics. In all cases, the values for the release rate constant (k) in all models were higher for the systems studied at 37 °C and pH 5 without irradiation as compared with those that were irradiated intermittently with an NIR laser (at 808 nm). Values of *n* obtained by the Peppas model can be used to judge the drug’s release mechanism [34,35]; if the *n* value is lower than 0.45, the kinetics of drug release from hydrogels correspond to a drug release mechanism controlled by Fickian diffusion.

If the drug is released through a porous material, the *n* value will be less than 0.5 due to the combination of partial diffusion mechanisms through a swollen matrix and through water-filled pores.

The obtained *n* values denote another simultaneous diffusion process; in thermosensitive polymers, it is attributed to the shrinkage of the networks due to the action of temperature [25]. The analysis of the 5FU release confirms the potential of these materials as a platform for topical-controlled drug delivery for skin cancer treatment.

## 4. Conclusions

A series of NVCL-based hydrogels were easily prepared by a photochemical method using a DVA crosslinker, NVP and/or LAMA comonomers, and IRGACURE 650^®^ as a photoinitiator. The hydrogel swelling in water at room temperature increased as the percentage of NVP or LAMA comonomers increased, and also its elasticity increased. The Q-values were between 2.9 and 4.1. The VPTT for all hydrogels was between 34 and 40 °C; these values increased with the content of NVP. The dry properties of the hydrogels showed also an increase in elasticity with the addition of NVP. All these are desirable basic properties of a hydrogel material intended for the treatment of skin malignancies by topical drug delivery. The hydrogels were loaded with gold nanorods and the antineoplastic drug 5-fluorouracil. The drug loading was between 3 and 5 wt%, the 5FU encapsulation efficiency was from 47 to 53 wt%, and the GNRDs encapsulation was from 3 to 6 wt%. The release behavior under skin-mimicking conditions showed fast release under normal temperatures, while NIR-irradiated samples moderated the release, possibly forming a skin layer on the hydrogel surface. The kinetics of drug release followed a Fickian diffusion mechanism, as demonstrated by the linear fitting by the Peppas model (*n* ≤ 0.45). These nanocomposite hydrogels have potential to be used in the future in a clinical setting to treat skin cancer through dual chemo-phototherapy treatment. More experiments toward this goal are considered for future investigations.

## Figures and Tables

**Table 1:** Composition, swelling degree, and VPTT in water for NVCL-based hydrogels.

Sample	LAMA Content ^(a)^ (wt%)	NVP Content ^(a)^ (mol%)	Q at 25 °C	Q at 37 °C	VPTT (°C)
H	-	-	3.4 ± 0.07	2.1 ± 0.02	37.0
HL1	1	-	3.7 ± 0.03	2.2 ± 0.00	36.5
HL2	2	-	3.9 ± 0.03	2.3 ± 0.06	36.0
HL3	3	-	4.0 ± 0.09	3.4 ± 0.02	34.0
HV5	-	5	3.8 ± 0.19	2.4 ± 0.07	38.5
HV10	-	10	4.1 ± 0.06	2.7 ± 0.06	39.0
HV15	-	15	4.2 ± 0.02	3.0 ± 0.02	40.0
HL3V5	3	5	3.6 ± 0.04	2.5 ± 0.02	40.0
HL3V10	3	10	3.7 ± 0.12	2.6 ± 0.13	40.0
HL3V15	3	15	4.1 ± 0.02	2.9 ± 0.03	40.0

^(a)^ With respect to NVCL.

**Table 2:** Mechanical results of hydrogel compression under submersion in water.

Sample	Young’s Modulus ^(a)^ (kPa) at 25 °C	Strain (%)	Young’s modulus ^(a)^ (kPa) at 37 °C	Strain (%)
H	114 ± 6	23 ± 1.5	142 ± 12	41 ± 2.0
HL1	60 ± 2	34 ± 0.4	127 ± 27	35 ± 2.3
HL2	49 ± 4	37 ± 0.4	85 ± 13	42 ± 0.4
HL3	43 ± 1	38 ± 1.2	91 ± 16	39 ± 2.0
HV5	93 ± 4	31 ± 1.0	107 ± 7	33 ± 3.0
HV10	84 ± 5	28 ± 1.0	89 ± 9	36 ± 1.2
HV15	76 ± 1	29 ± 0.6	120 ± 20	32 ± 0.7
HL3V5	93 ± 9	29 ± 0.7	114 ± 1	38 ± 1.0
HL3V10	92 ± 4	30 ± 2.5	287 ± 19	27 ± 2.3
HL3V15	78 ± 6	30 ± 0.7	191 ± 37	32 ± 2.7

^(a)^ Moduli were measured as SEC line at 5% of strain.

**Table 3:** Data of 5FU/GNRDs loading and encapsulation efficiency in hydrogels.

Hydrogel	5FU Loading (wt%)	Encapsulation Efficiency (%)	GNRD Loading (wt%)
H	4.03 ± 0.4	51.48 ± 0.3	3.78 ± 0.3
HL3	2.99 ± 0.0	47.50 ± 0.3	4.65 ± 0.9
HV15	3.92 ± 0.3	51.09 ± 3.2	4.12 ± 0.1
HL3V15	4.93 ± 1.3	52.50 ± 10.2	5.66 ± 0.2

**Table 4:** The 5FU in vitro release kinetics: data fitted with different models.

Hydrogel	Model	Release Rate (k)		Regression Coefficient (r^2^)	
		Not Irradiated	Irradiated	Not Irradiated	Irradiated
H	Zero order	0.0002	0.0002	0.58	0.86
	First order	0.0004	0.0004	0.59	0.86
	Higuchi	0.0111	0.0050	0.69	0.87
	Peppas	0.3575,	0.2876,	0.76	0.86
		n = 0.09	n = 0.09		
HL3	Zero order	0.0006	0.0004	0.64	0.53
	First order	0.0009	0.0006	0.76	0.55
	Higuchi	0.0092	0.0047	0.83	0.58
	Peppas	0.3715,	0.2985,	0.90	0.78
		n = 0.09	n = 0.09		
HV15	Zero order	0.0007	0.0006	0.96	0.88
	First order	0.0013	0.0009	0.96	0.89
	Higuchi	0.0096	0.0081	0.97	0.93
	Peppas	0.3956,	0.2755,	0.61	0.73
		n = 0.04	n = 0.05		
HL3V15	Zero order	0.0009	0.0005	0.90	0.67
	First order	0.0008	0.0007	0.68	0.64
	Higuchi	0.0079	0.0062	0.75	0.73
	Peppas	0.3822,	0.2817,	0.82	0.77
		n = 0.05	n = 0.05		

## Data Availability

The data presented in this study are available on request from the corresponding authors.

## Supplementary Materials

The following supporting information can be downloaded at: https://www.mdpi.com/article/10.3390/pharmaceutics15041097/s1: Figure S1. Equilibrium swelling degree (Q) as function of temperature of HG-NVCL-L1, Figure S2. Equilibrium swelling degree (Q) as function of temperature of HG-NVCL-L2, Figure S3. Equilibrium swelling degree (Q) as function of temperature of HG-NVCL-VP5, Figure S4. Equilibrium swelling degree (Q) as function of temperature of HG-NVCL-VP10, Figure S5. Equilibrium swelling degree (Q) as function of temperature of HG-NVCL-L3-VP5, Figure S6. Equilibrium swelling degree (Q) as function of temperature of HG-NVCL-L3-VP10, Figure S7. DSC thermograms of NVCL-based hydrogels copolymerized with LAMA, Figure S8. DSC thermograms of NVCL-based hydrogels copolymerized with NVP, Figure S9. Stress–strain compression curves under submersion in water at 37 °C, for NVCL hydrogels with LAMA, Figure S10. Stress–strain compression curves under submersion in water at 37 °C, for NVCL hydrogels with NVP, Figure S11. Stress–strain compression curves under submersion in water at 37 °C, for NVCL hydrogels with LAMA and NVP, Figure S12. TEM micrography of colloidal gold nanorods with aspect ratio of 4.2, Figure S13. Thermogravimetric analysis of hydrogel H, Figure S14. Thermogravimetric analysis of hydrogel HL3, Figure S15. Thermogravimetric analysis of hydrogel HV15, Figure S16. Thermogravimetric analysis of hydrogel HL3V15, Figure S17. Thermogravimetric analysis of hydrogel H loaded with 5FU and GNRDs, Figure S18. Thermogravimetric analysis of hydrogel HL3 loaded with 5FU and GNRDs, Figure S19. Thermogravimetric analysis of hydrogel HV15 loaded with 5FU and GNRDs, Figure S20. Thermogravimetric analysis of hydrogel HL3V15 loaded with 5FU and GNRDs, Figure S21. FT-IR spectrum for hydrogels H, HL3, HV15, and HL3V15, Figure S22. UV–Vis spectrum of 5FU, Figure S23. Cumulative 5FU release from PNVCL hydrogel (H), containing only 5FU, with and without NIR irradiation.

## Abbreviations

5FU	5-fluorouracil	n	diffusional exponent
AA	ascorbic acid	NIR	near-infrared region
CTAB	cetyltrimethylammonium bromide	NVCL	*N*-vinylcaprolactam
DL	drug loading	NVP	*N*-vinylpyrrolidone
DMA	dynamic mechanical analysis	PBS	phosphate-buffered saline
DR	drug release	PNVCL	poly(*N*-vinylcaprolactam)
DSC	differential scanning calorimetry	*Q*	swelling ratio
EE	encapsulation efficiency	T_g_	glass transition temperature
*F*	fractional drug release	TGA	thermogravimetric analysis
GNRDs	gold nanorods	UV–Vis	UV–Vis spectroscopy
IRGACURE 651^®^	2,2-dimethoxy-2-phenylacetophenone	*V_d_*	volume of dry hydrogel
*k*	release rate constant	VPTT	volume phase transition temperature
LAMA	2-lactobionamidoethyl methacrylate	*V_s_*	volume of swelled hydrogel
LSPR	localized surface plasmon resonance	*W_d_*	dry hydrogel mass
*M*_0_	initial mass of drug	*W_s_*	swelled hydrogel mass
MD	mass of drug	*W_sol_*	weight of the absorbed solvent
MD_h_	mass of drug in the hydrogel	*ρ_d_*	density of the dry hydrogel
*M_t_*	mass of drug at time *t*	*ρ_so_*	density of the solvent
