# Supplementary material for: Poly(N-vinylcaprolactam)–Gold Nanorods–5 Fluorouracil Hydrogels: In the Quest of a Material for Topical Therapies against Melanoma Skin Cancer
Source: Pharmaceutics. 2023 Mar 29;15(4):1097. doi: 10.3390/pharmaceutics15041097 (PMC10145490; doi:10.3390/pharmaceutics15041097)
Supplement: Supplementary file 1 [file pharmaceutics-15-01097-s001.zip › pharmaceutics-2240448-supplementary.pdf]

Supplementary material

# Poly(*N*-Vinylcaprolactam)–Gold Nanorods–5 Fluorouracil Hydrogels: In the Quest of a Material for Topical Therapies against Melanoma Skin Cancer

**Mirian A. González-Ayón *, Alondra Rochin-Galaviz, Arturo Zizumbo-López and Angel Licea-Claverie ***

Centro de Graduados e Investigación en Química, Tecnológico Nacional de México/ Instituto Tecnológico de Tijuana, Apartado Postal 1166, Tijuana 22454, Mexico

* Correspondence: mirian.gonzalez@tectijuana.edu.mx (M.A.G.-A.); aliceac@tectijuana.mx (A.L.-C.)

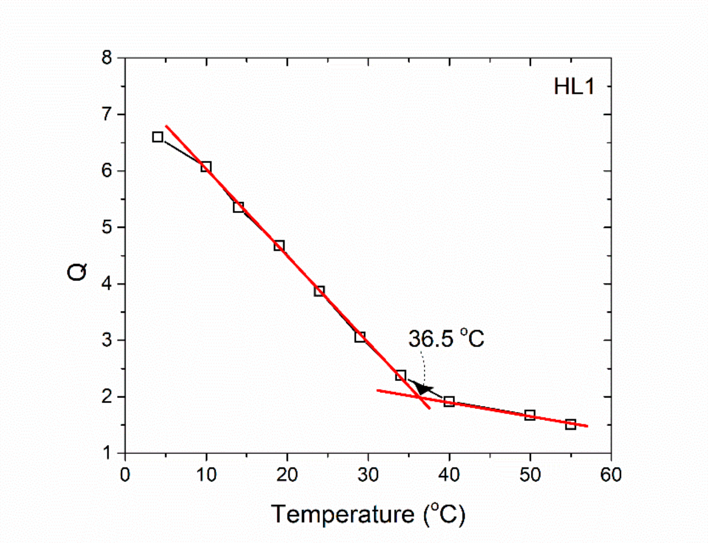


**Figure S1.** Equilibrium swelling degree (Q) as function of temperature of HG-NVCL-L1.

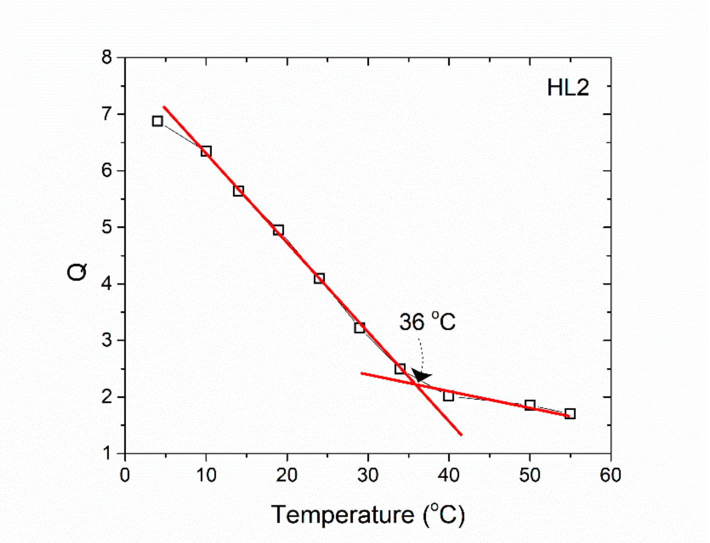


**Figure S2.** Equilibrium swelling degree (Q) as function of temperature of HG-NVCL-L2.

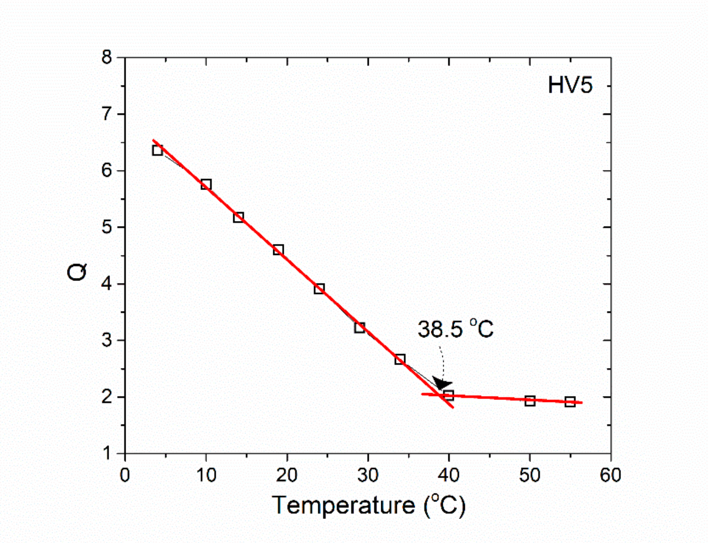


**Figure S3.** Equilibrium swelling degree (Q) as function of temperature of HG-NVCL-VP5.

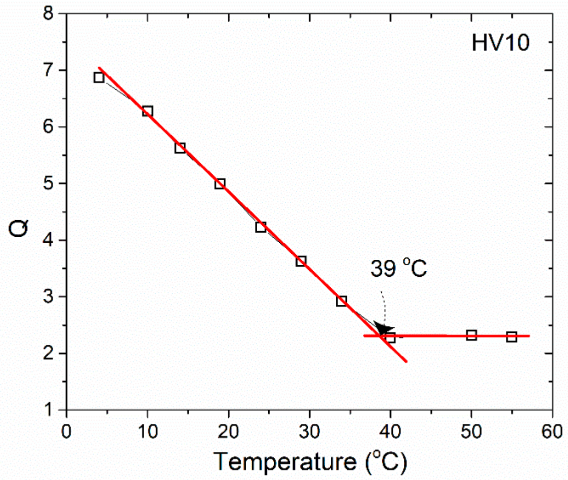


**Figure S4.** Equilibrium swelling degree (Q) as function of temperature of HG-NVCL-VP10.

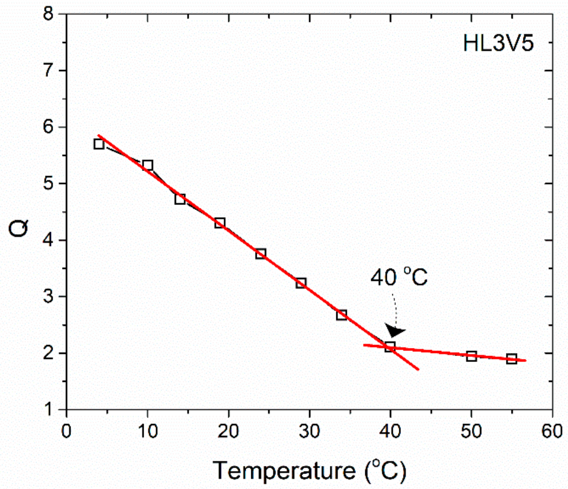


**Figure S5.** Equilibrium swelling degree (Q) as function of temperature of HG-NVCL-L3-VP5.

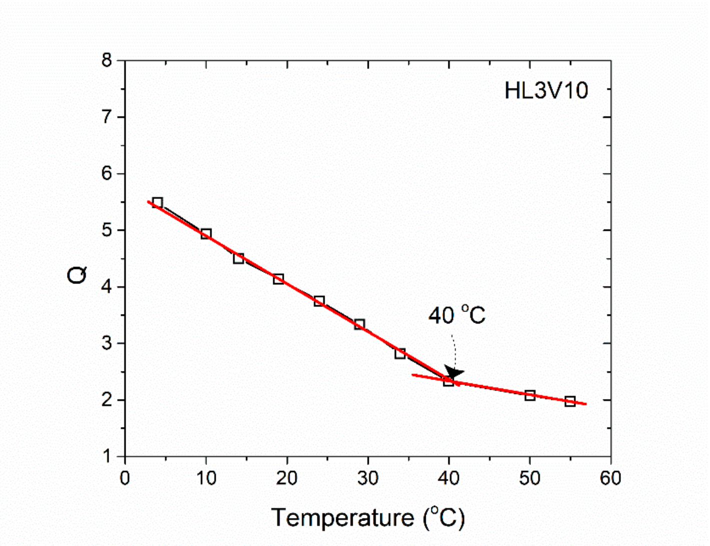


**Figure S6.** Equilibrium swelling degree (Q) as function of temperature of HG-NVCL-L3-VP10.

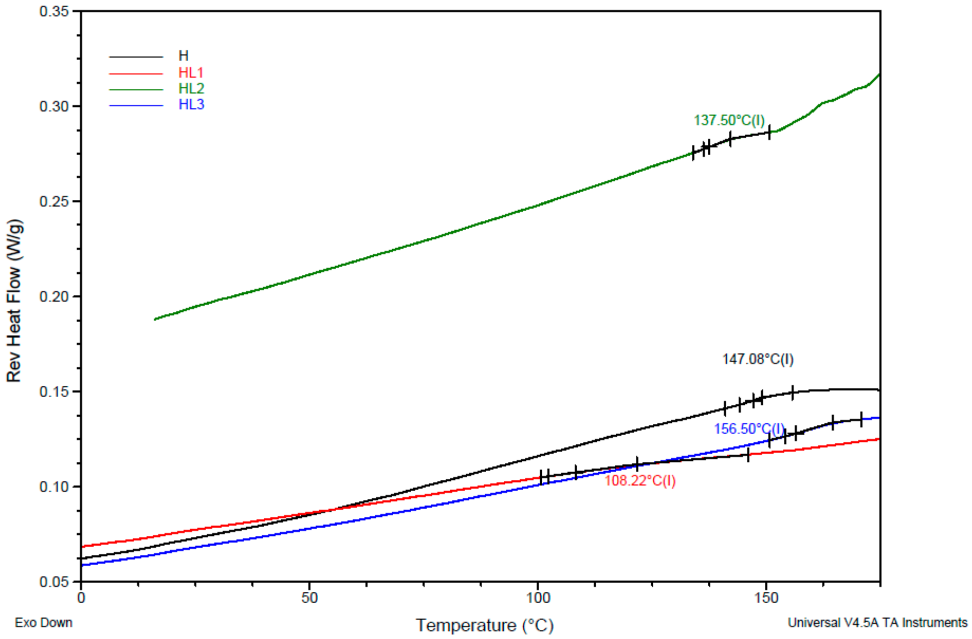


**Figure S7.** DSC thermograms of NVCL-based hydrogels copolymerized with LAMA.

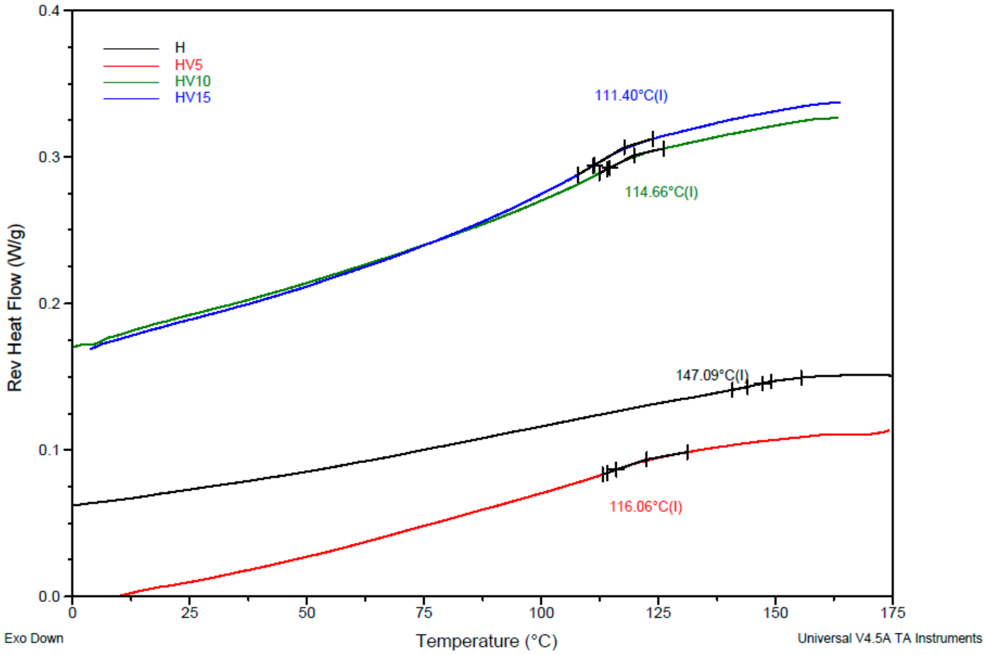


**Figure S8.** DSC thermograms of NVCL-based hydrogels copolymerized with NVP.

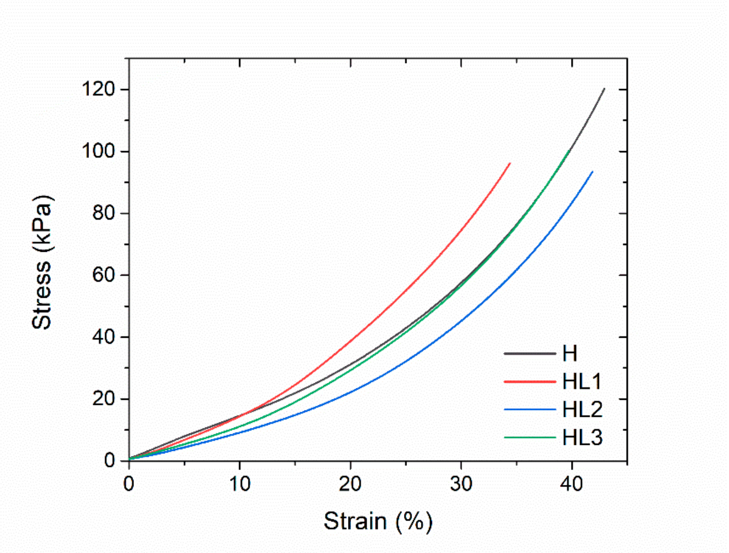


**Figure S9.** Stress-strain compression curves under submersion in water at 37 °C, for NVCL-hydrogels with LAMA.

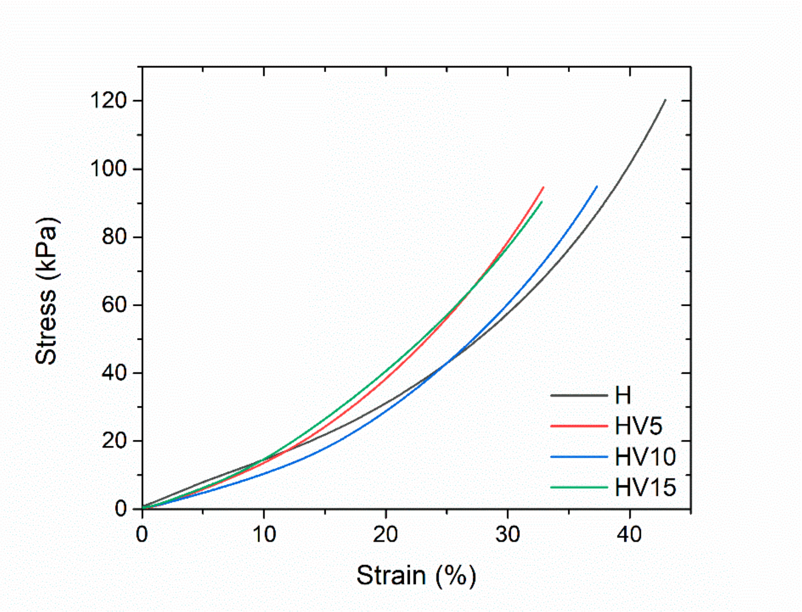


**Figure S10.** Stress-strain compression curves under submersion in water at 37 °C, for NVCL-hydrogels with NVP.

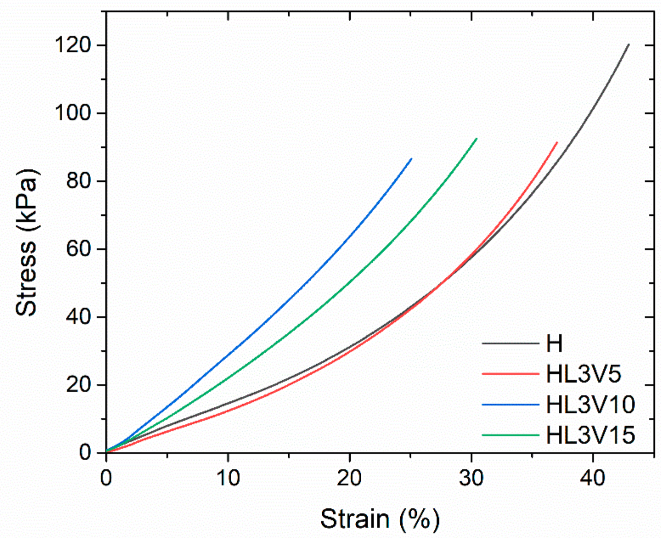


**Figure S11.** Stress-strain compression curves under submersion in water at 37 °C, for NVCL-hydrogels with LAMA and NVP.

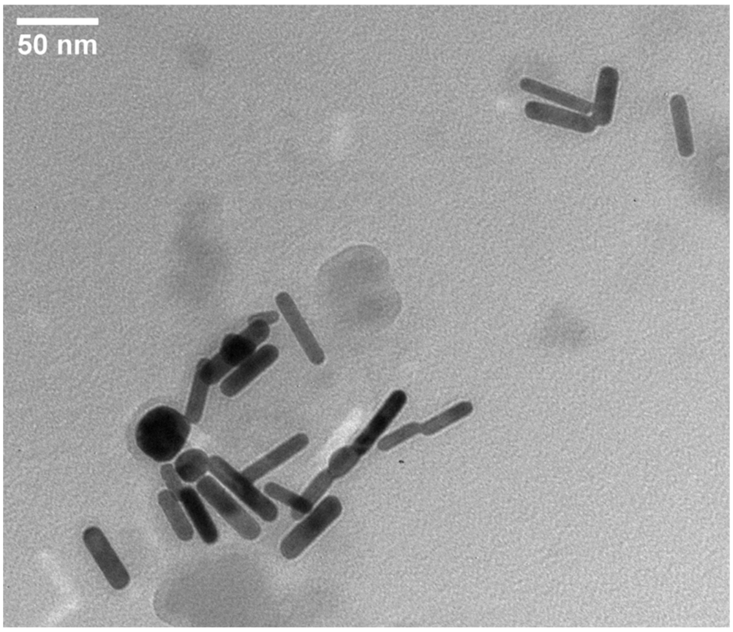


**Figure S12**. TEM micrography of colloidal gold nanorods of aspect ratio of 4.2.

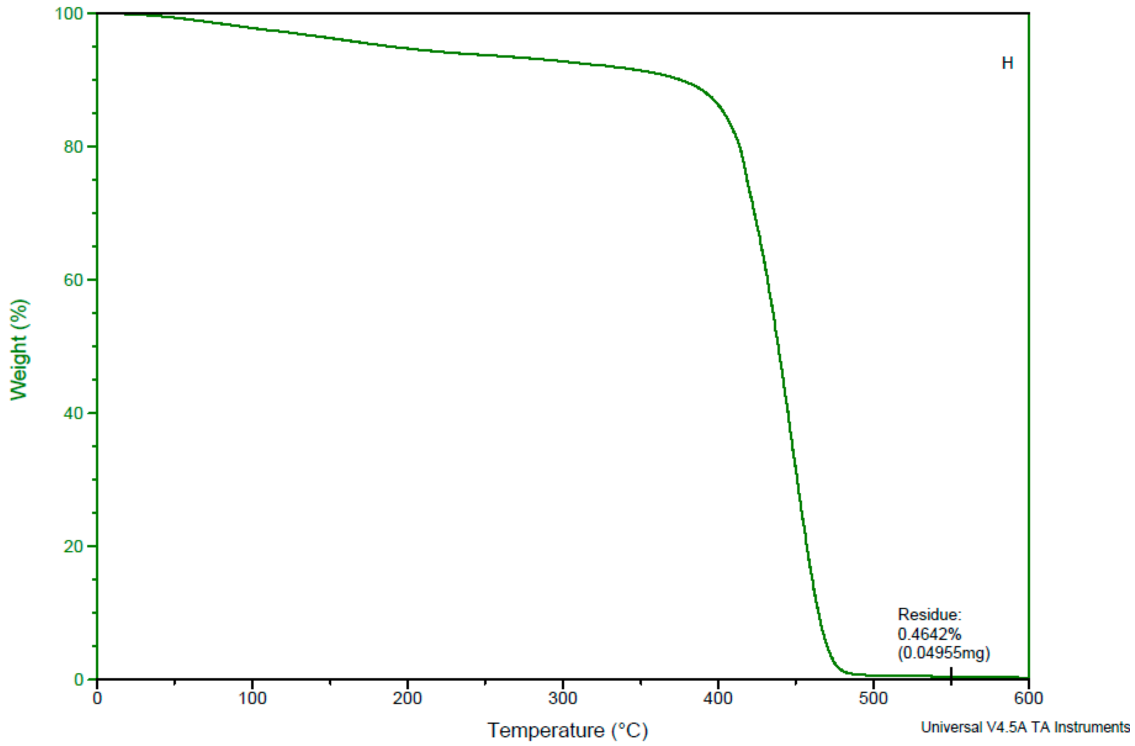


**Figure S13.** Thermogravimetric analysis of hydrogel H.

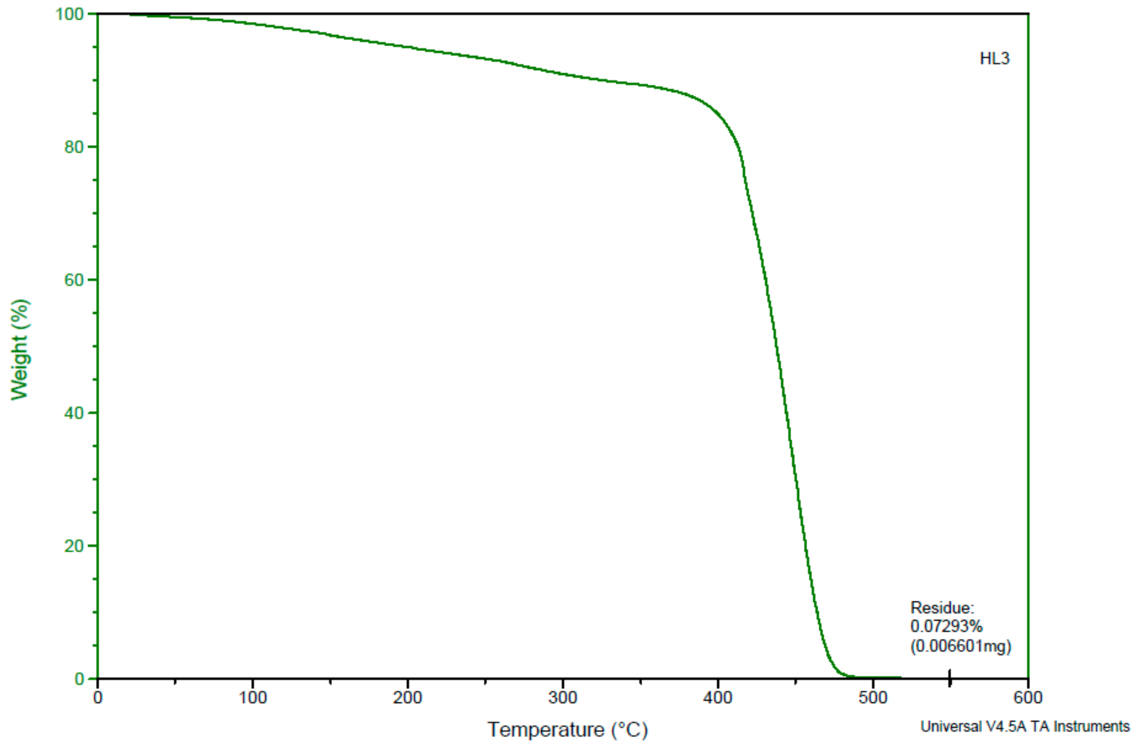


**Figure S14.** Thermogravimetric analysis of hydrogel HL3.

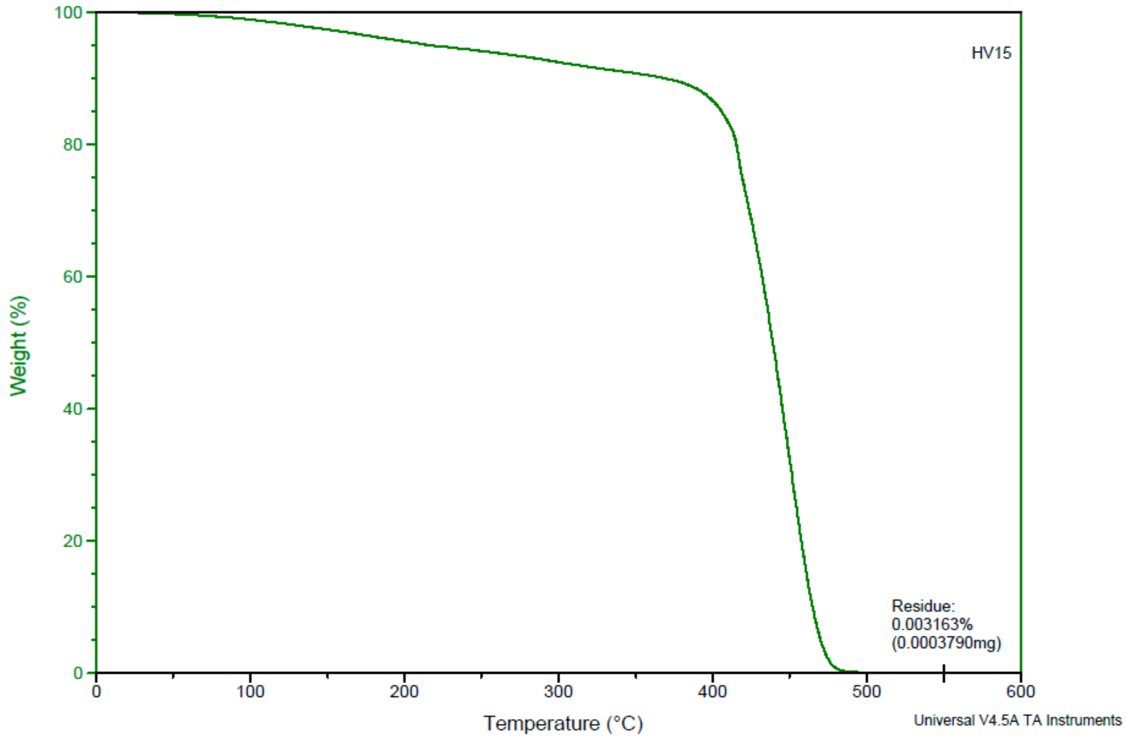


**Figure S15.** Thermogravimetric analysis of hydrogel HV15.

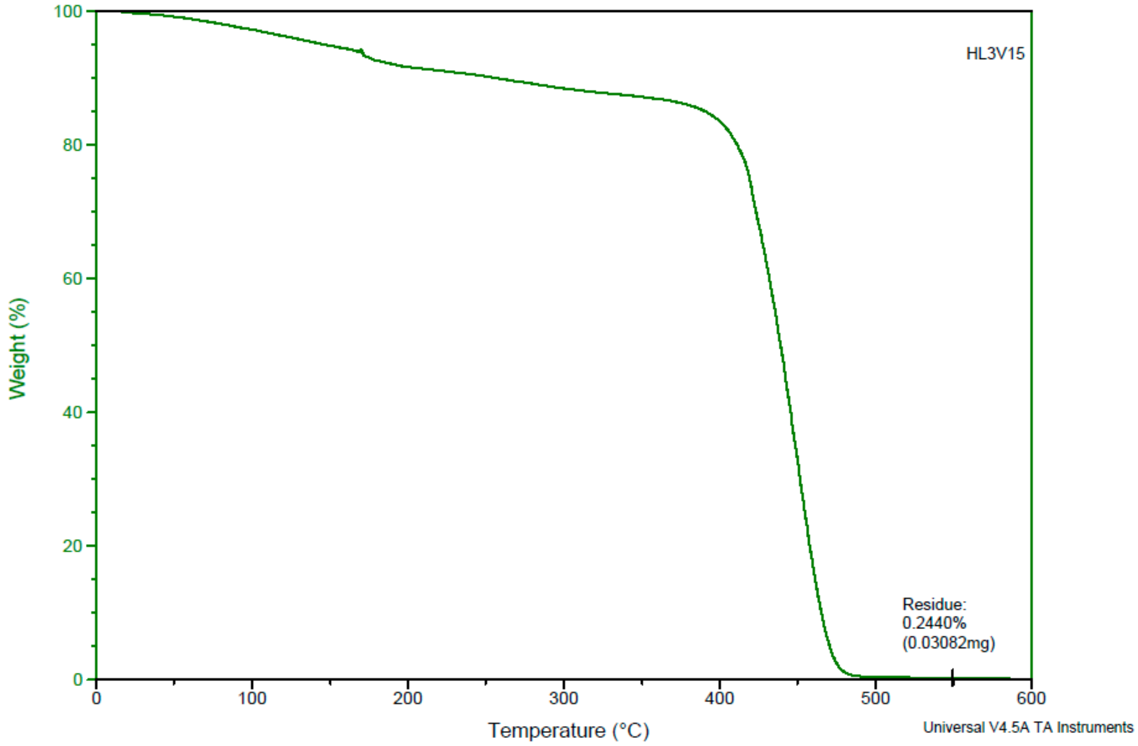


**Figure S16.** Thermogravimetric analysis of hydrogel HL3V15.

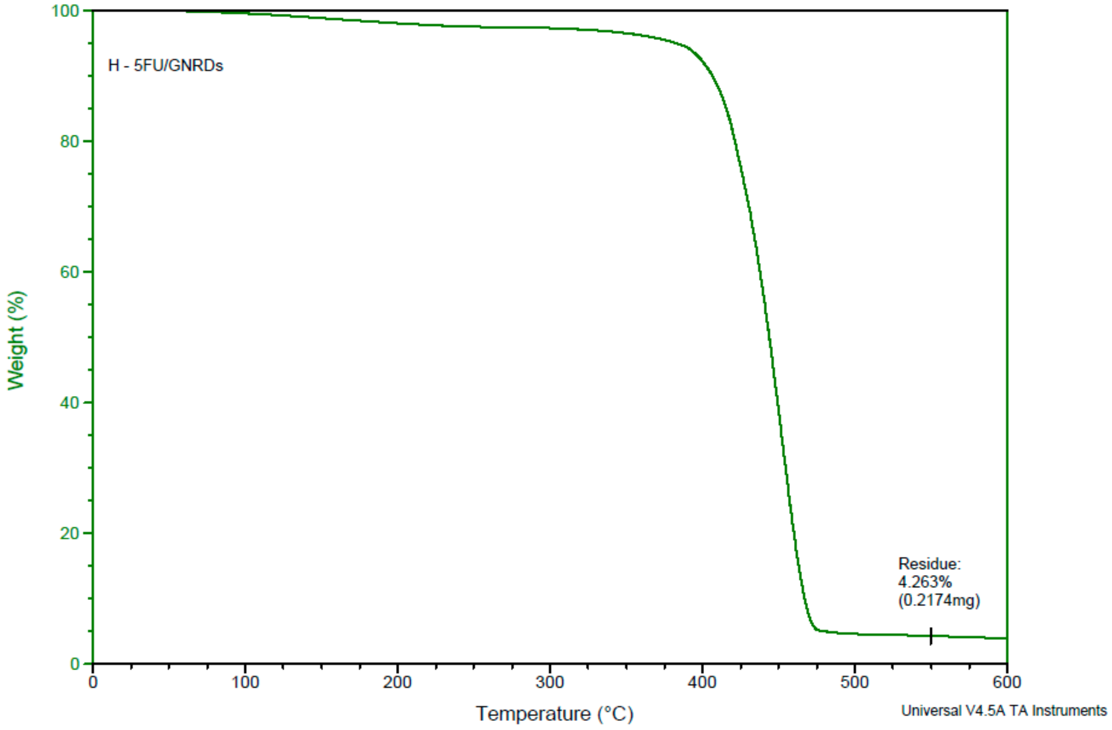


**Figure S17.** Thermogravimetric analysis of hydrogel H loaded with 5FU and GNRDs.

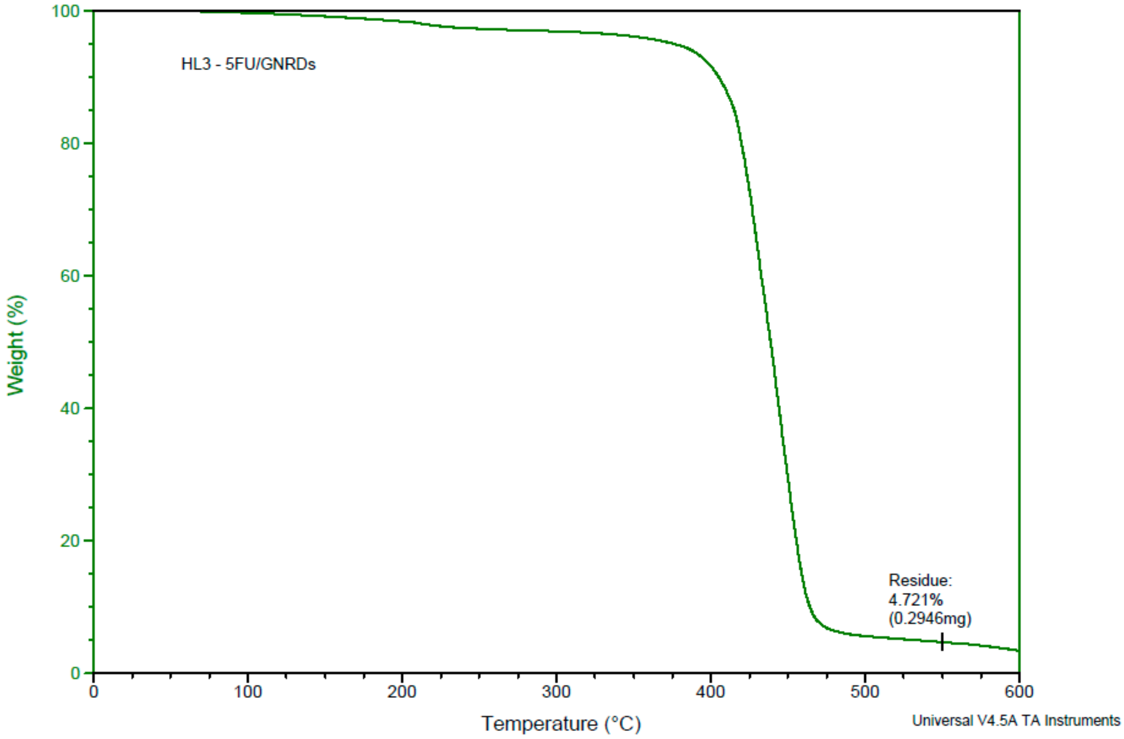


**Figure S18.** Thermogravimetric analysis of hydrogel HL3 loaded with 5FU and GNRDs.

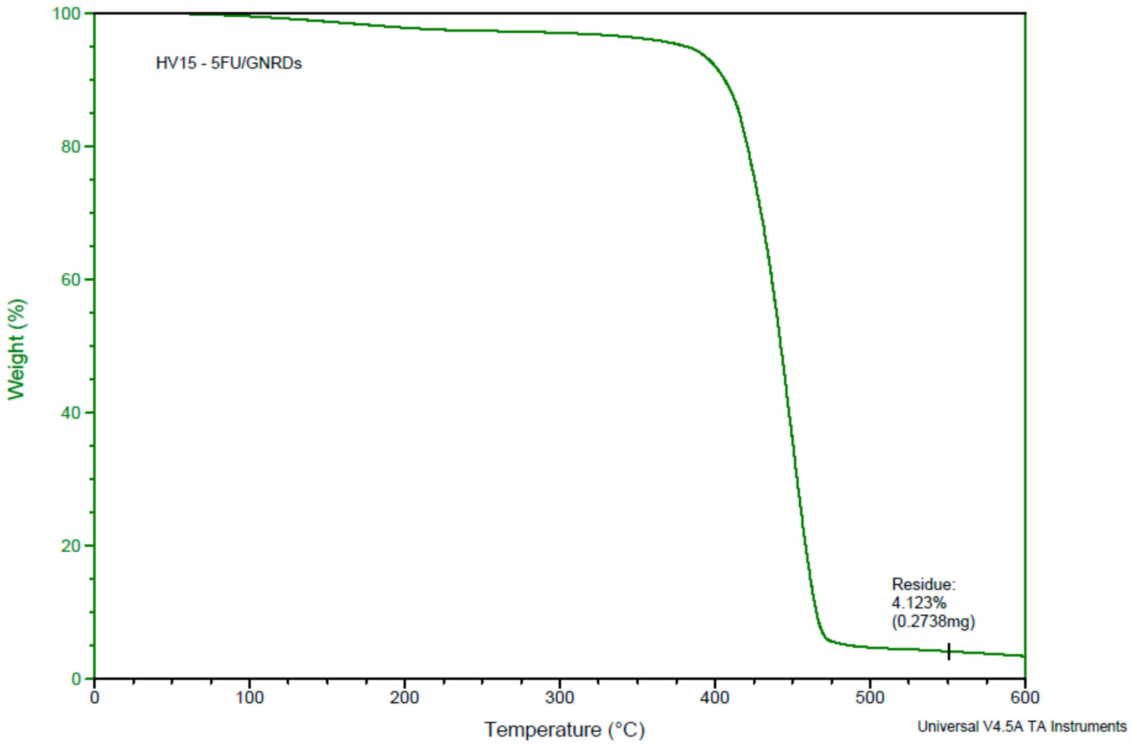


**Figure S19.** Thermogravimetric analysis of hydrogel HV15 loaded with 5FU and GNRDs.

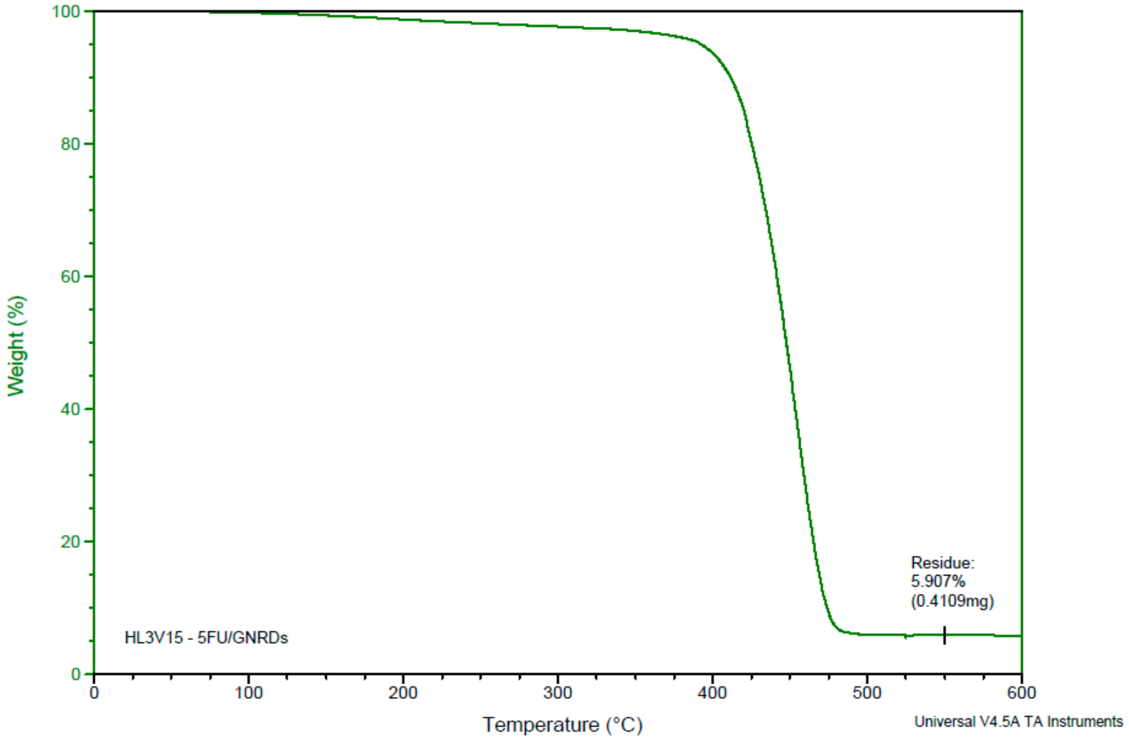


**Figure S20.** Thermogravimetric analysis of hydrogel HL3V15 loaded with 5FU and GNRDs.

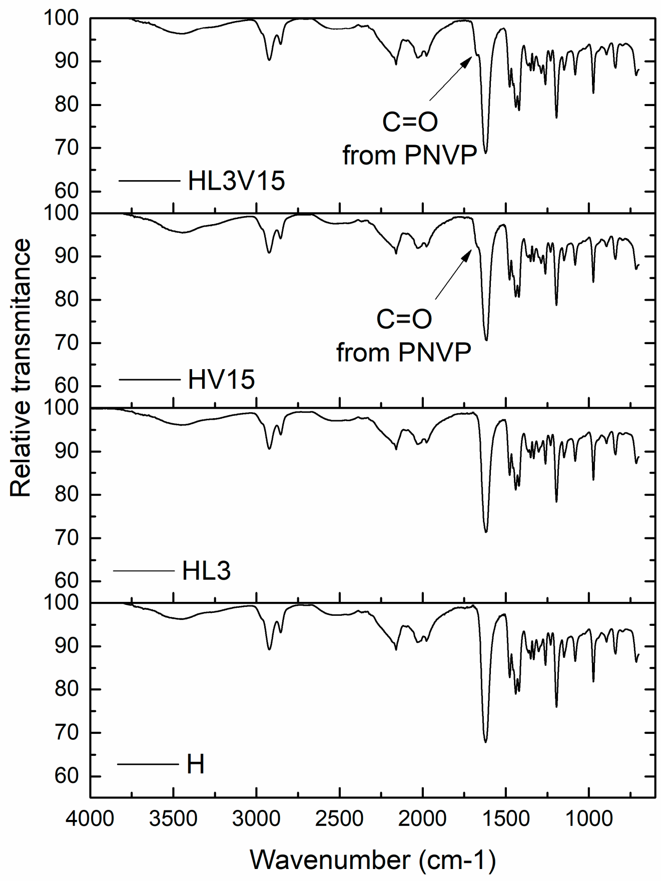


**Figure S21.** FT-IR spectrum for hydrogels: H, HL3, HV15 and HL3V15.

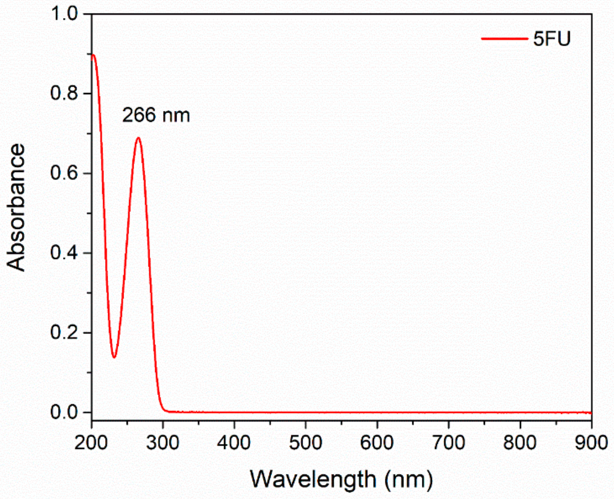


**Figure S22.** UV-Vis spectrum of 5FU.

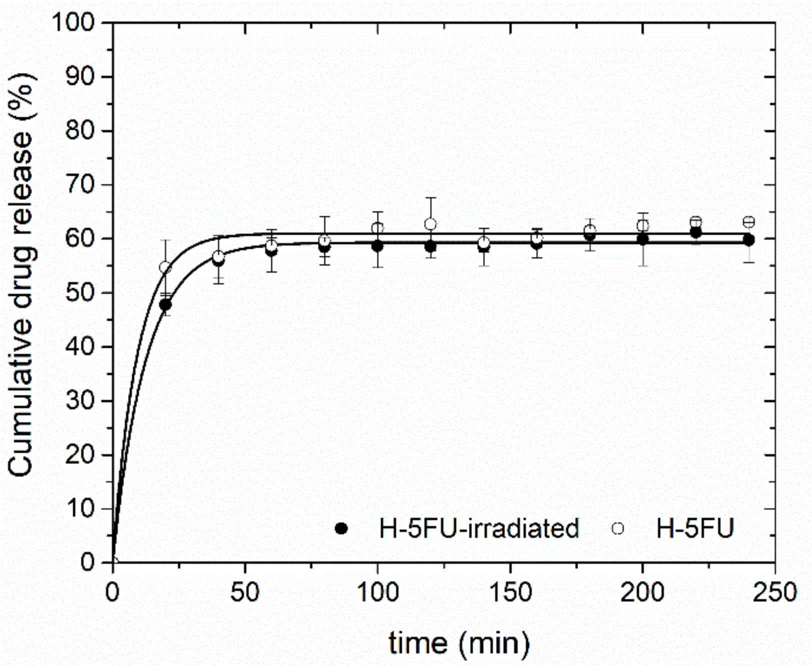


**Figure S23.** Cumulative 5FU release form PNVCL hydrogel (H), containing only 5FU, with and without NIR irradiation.
